# Sulforaphane Enhanced Proliferation of Porcine Satellite Cells via Epigenetic Augmentation of *SMAD7*

**DOI:** 10.3390/ani12111365

**Published:** 2022-05-26

**Authors:** Rui Zhang, Christiane Neuhoff, Qin Yang, Mehmet U. Cinar, Muhammad J. Uddin, Ernst Tholen, Karl Schellander, Dawit Tesfaye

**Affiliations:** 1 Meat Processing Key Laboratory of Sichuan Province, College of Food and Biological Engineering, Chengdu University, Chengdu 610106, China; zhangrui@cdu.edu.cn; 2 Animal Breeding and Husbandry Group, Institute of Animal Science, University of Bonn, 53115 Bonn, Germany; christiane.neuhoff@hs-gm.de (C.N.); qyan@itw.uni-bonn.de (Q.Y.); mucinar@erciyes.edu.tr (M.U.C.); jasim.uddin@murdoch.edu.au (M.J.U.); etho@itw.uni-bonn.de (E.T.); ksch@itw.uni-bonn.de (K.S.); 3 Department of Animal Science, Faculty of Agriculture, Erciyes University, Kayseri 38030, Turkey; 4 Department of Veterinary Microbiology and Pathology, College of Veterinary Medicine, Washington State University, Pullman, WA 99164, USA; 5 College of Veterinary Medicine, Murdoch University, Murdoch 6150, Australia; 6 Animal Reproduction and Biotechnology Laboratory, Department of Biomedical Sciences, Colorado State University, Fort Collins, CO 80523, USA

**Keywords:** pig, sulforaphane, muscle growth, skeletal muscle stem cell, histone acetylation, microRNA

## Abstract

**Simple Summary:**

In livestock agriculture, increasing muscle growth and meat output has been a continual hot subject. A few natural compounds have proved to have the ability to accelerate muscle growth. Sulforaphane (SFN), abundant in cruciferous vegetables, has previously been shown to enhance skeletal muscle growth. In this work, we found that SFN could activate the skeletal muscle stem cell amplification. Furthermore, the underlying mechanisms were investigated. This work contributes to our understanding of how organisms interact with environmental cues such as nutrition and indicates a potential technique to boost animal output by managing feed. Meanwhile, our research is taking us one step closer to understanding how green vegetables, such as broccoli, enhance fitness and health while reducing calorie intake.

**Abstract:**

Satellite cells take an indispensable place in skeletal muscle regeneration, maintenance, and growth. However, only limited works have investigated effects of dietary compounds on the proliferation of porcine satellite cells (PSCs) and related mechanisms. Sulforaphane (SFN) at multiple levels was applied to PSCs. The PSCs’ viability and HDAC activity were measured with a WST-1 cell proliferation kit and Color-de-Lys^®^ HDAC colorimetric activity assay kit. Gene expression and epigenetics modification were tested with qRT-PCR, Western blot, bisulfite sequencing, and ChIP-qPCR. This study found that SFN enhanced PSC proliferation and altered mRNA expression levels of myogenic regulatory factors. In addition, SFN inhibited histone deacetylase (HDAC) activity, disturbed mRNA levels of HDAC family members, and elevated acetylated histone H3 and H4 abundance in PSCs. Furthermore, both mRNA and protein levels of the Smad family member 7 (*SMAD7*) in PSCs were upregulated after SFN treatment. Finally, it was found that SFN increased the acetylation level of histone H4 in the *SMAD7* promoter, decreased the expression of microRNAs, including *ssc-miR-15a*, *ssc-miR-15b*, *ssc-miR-92a*, *ssc-miR-17-5p*, *ssc-miR-20a-5p*, and *ssc-miR-106a*, targeting *SMAD7*, but did not impact on the *SMAD7* promoter’s methylation status in PSCs. In summary, SFN was found to boost PSC proliferation and epigenetically increase porcine *SMAD7* expression, which indicates a potential application of SFN in modulation of skeletal muscle growth.

## 1. Introduction

Satellite cells are skeletal muscle stem cells that are found between the plasma membrane and the basal lamina of myofibers, where they are kept in a mitotically quiescent condition until they are activated to form myoblasts [1]. Myoblasts are incorporated into existing myofibers or other myoblasts as myogenesis progresses, supporting muscle growth and regeneration. Myonuclei accumulation is closely related to the increase in myofiber size and muscle mass [2]. Multiple components, such as myogenic regulatory factors and myocyte enhancer factor 2 (MEF2) family members, help to organize and drive myogenesis [3]. Porcine satellite cells (PSCs) serve as a good model to study skeletal muscle stem cell activity [4].

SMAD family member 7 (*SMAD7*) is an important regulator of skeletal muscle development and regeneration by blocking transforming growth factor β (TGF-β) signaling [5]. In mice, overexpression of *SMAD7* reverses myostatin-induced skeletal muscle atrophy and causes skeletal muscular growth. [6]. *SMAD7*-null mice, on the other hand, have a deficiency in myoblast proliferation and have a low muscle mass [7]. Adeno-associated virus mediated *SMAD7* administration alleviates cancer-related muscular atrophy in mice [6]. *SMAD7* also can induce myogenesis by interacting directly with MYOD in the nucleus [8]. As a critical modulator of TGF-β signaling, the expression and stability of *SMAD7* are modulated in multiple ways, including lysine residue acetylation [9], promoter methylation [10], and microRNA (miRNA) post-transcriptional regulation [11].

Sulforaphane (SFN) suppresses histone deacetylase (HDAC) activity while also being a strong inducer of phase II enzyme via nuclear factor E2-related factor-2 (NFE2L2, also termed Nrf2) signaling [12]. SFN shows a lot of promise as a cancer chemoprevention agent. Meanwhile, diet supplemented with SFN is shown to suppress the delayed onset muscle soreness in humans through Nrf2 activation [13], increases the amount of skeletal muscle satellite cell for muscle homeostasis, hypertrophy, or repair in mice [14], and alleviates muscle atrophy through the Nrf2 and Akt/Foxo1 pathway [15,16]. Furthermore, in recent years, the potential use of SFN in the agriculture business has gained a lot of attention [4,17]. As previously established, SFN epigenetically decreases myostatin expression in PSCs [4]. However, there have been few investigations on the impact of SFN on PSCs. The current study looks into the influence of SFN on PSCs as well as the underlying processes.

## 2. Materials and Methods

### 2.1. Porcine Skeletal Muscle Satellite Cell Culture

**Figure 1:**
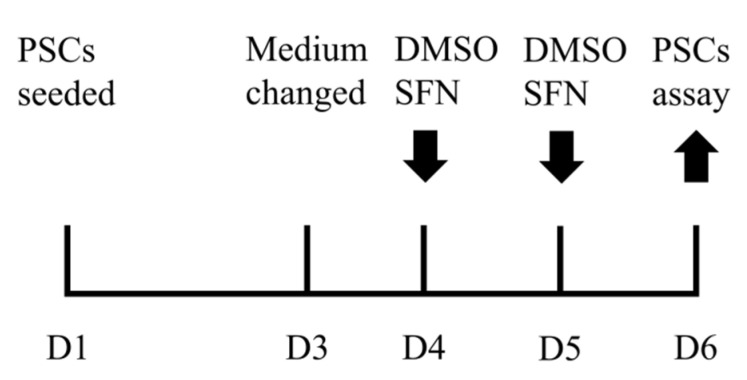
PSC culture and SFN treatment procedure.

The semimembranosus muscles of 21 days old purebred Pietrain male piglets were used for PSC isolation, where Pietrain was selected mainly due to the breed advantages in the growth performance, body composition, and muscle microstructure [18,19]. The isolation of PSCs followed the method detailed in our last report [4]. Briefly, the isolated PSCs were seeded in cell culture plate at Day 1 (D1). Cell culture medium was changed at D3 and then refreshed every day. PSCs were treated with DMSO vehicle control (Ctrl) or SFN (5, 10, 15, or 20 μM, LKT, Hamburg, Germany) dissolved in DMSO at D4 and D5 with three independent assays for each treatment, and harvested at D6 for multiple PSC assays, as shown in Figure 1.

### 2.2. PSC Viability Assay

The viability of PSCs treated by DMSO or SFN (5, 10, 15, or 20 μM) in 96-well plates was determined using a WST-1 cell proliferation assay kit (Cayman Chemical, Hamburg, Germany). For each well, 10 µL WST-1 reagent was added and cells were cultured at 37 °C for two hours. The absorbance at 450 nm was assessed and all readouts were normalized with the background value.

### 2.3. HDAC Activity Assay

HDAC activity was assessed with a Color-de-Lys^®^ HDAC colorimetric activity assay kit (Enzo Life Science, Lörrach, Germany). PSC nuclear extract was mixed with 25 µL assay buffer, 25 µL 37 °C pre-warmed Color-de-Lys^®^ substrate was added, and the mixture was incubated at 37 °C for one hour. Then, 50 µL of Color-de-Lys^®^ developer containing 2 µM Trichostatin A was added to stop the reaction followed by 37 °C incubation for 15 min. The results of absorbance reading at 405 nm wavelength were analyzed after being normalized to a no-enzyme control.

### 2.4. MiRNAs Targeting Porcine SMAD7

TargetScan [20] was used to predict miRNAs targeting porcine *SMAD7*. The predicted interaction between miRNA and 3′ UTR of porcine *SMAD7* was further verified by RNAHybrid [21] and RNA22 [22].

### 2.5. Quantitative Real-Time PCR (qRT-PCR) of mRNA and miRNA

Total RNA including miRNA was isolated using an AllPrep DNA/RNA/miRNA Universal Kit (Qiagen, Hilden, Germany). A First Strand cDNA Synthesis Kit (Thermo Scientific, Dreieich, Germany) and miScript PCR Starter Kit (Qiagen, Hilden, Germany) were used to synthesis mRNA and miRNA cDNA, respectively. The qRT-PCR was carried out using iTaq SYBR Green Supermix (Bio-Rad, Feldkirchen, Germany) or miScript PCR Starter Kit (Qiagen, Hilden, Germany) to quantify expression levels of mRNA and miRNA, respectively. Primer3 was utilized to design all primers for qRT-PCR [23] and the primer sequences were shown in Table 1. The Ct value from qRT-PCR was analyzed using the 2^−ΔΔCt^ method [24], and hypoxanthine phosphoribosyltransferase 1 (HPRT1) and U6 small nuclear RNA were used as endogenous references for mRNA and miRNA, respectively.

### 2.6. Western Blot

Western blot was performed following our routine method [18]. The primary antibodies utilized in this work included these for acetyl-histone H4 (06-866, Millipore, Burlington, MA, USA), acetyl-histone H3 (06-599, Burlington, MA, USA), SMAD7 (sc-11392, Santa Cruz, Dallas, TX, USA), and TGFB1 (sc-146, Santa Cruz, Dallas, TX, USA). The applied secondary antibodies were goat anti-rabbit (sc-2004, Santa Cruz, Dallas, TX, USA) and donkey anti-goat (sc-2020, Santa Cruz, Dallas, TX, USA). The blot signals were visualized using Clarity Western ECL Substrate (Bio-Rad, Feldkirchen, Germany) and acquired by ChemiDoc XRS system (Bio-Rad, Feldkirchen, Germany). Coomassie-Brilliant Blue staining was used as a loading control.

### 2.7. Bisulfite Sequencing

Genomic DNA was isolated using an AllPrep DNA/RNA/miRNA Universal Kit (Qiagen, Hilden, Germany). The 2 kilobases (kb) (NC_010443.5:c98545924-98543925) 5′ upstream of the porcine *SMAD7* (Gene ID: 100521305) transcription start site were used for CpG island detection and bisulfite sequencing PCR primer design with aid of Methprimer [25]. Following the manual of the EZ DNA Methylation-Direct Kit (Zymo Research, Irvine, CA, USA), bisulfite treated PSCs’ genomic DNA was used as the template for PCR amplification. The amplicon was inserted into a pGEM T-easy vector (Promega, Heidelberg, Germany). At least six positive clones were sequenced using the sequencer CEQ8000 (Beckman Coulter, Miami, FL, USA). BiQ Analyzer was applied in bisulfite sequencing results analysis [26].

### 2.8. Chromatin Immunoprecipitation (ChIP) Assay

The ChIP assay was performed according to the manual of the ChIP Assay Kit (Millipore, Darmstadt, Germany) with acetyl-histone H4 antibody (06-866, Millipore, Burlington, MA, USA), which was followed by quantitative PCR (ChIP-qPCR) using porcine *SMAD7* promoter-specific primers (Table 1). ChIP assay performed with normal rabbit IgG (2729S, Cell Signaling Technology, Danvers, IL, USA) was taken for the negative control. According to the percentage input method, the qPCR signal obtained from the ChIP DNA with acetyl-histone H4 antibody was normalized with a signal obtained from the 5% input DNA sample: 100 × 2^(Ct of ChIP(input)—log_2_ 20—Ct of ChIP(acH4)). The ChIP-qPCR data are expressed as the relative enrichment fold change of the SFN group compared to the Ctrl group with three independent replications for each group.

### 2.9. Statistical Analysis

Student’s *t* test was applied to determine the statistical significance of differences between the Ctrl and SFN treatment groups. The data are expressed as mean ± standard error (SE) of three independent assays. * *p* < 0.05, ** *p* < 0.01, and *** *p* < 0.001 were utilized as levels of significance.

## 3. Results

### 3.1. SFN Modulated PSC Proliferation and Myogenic Genes Expression

**Figure 2:**
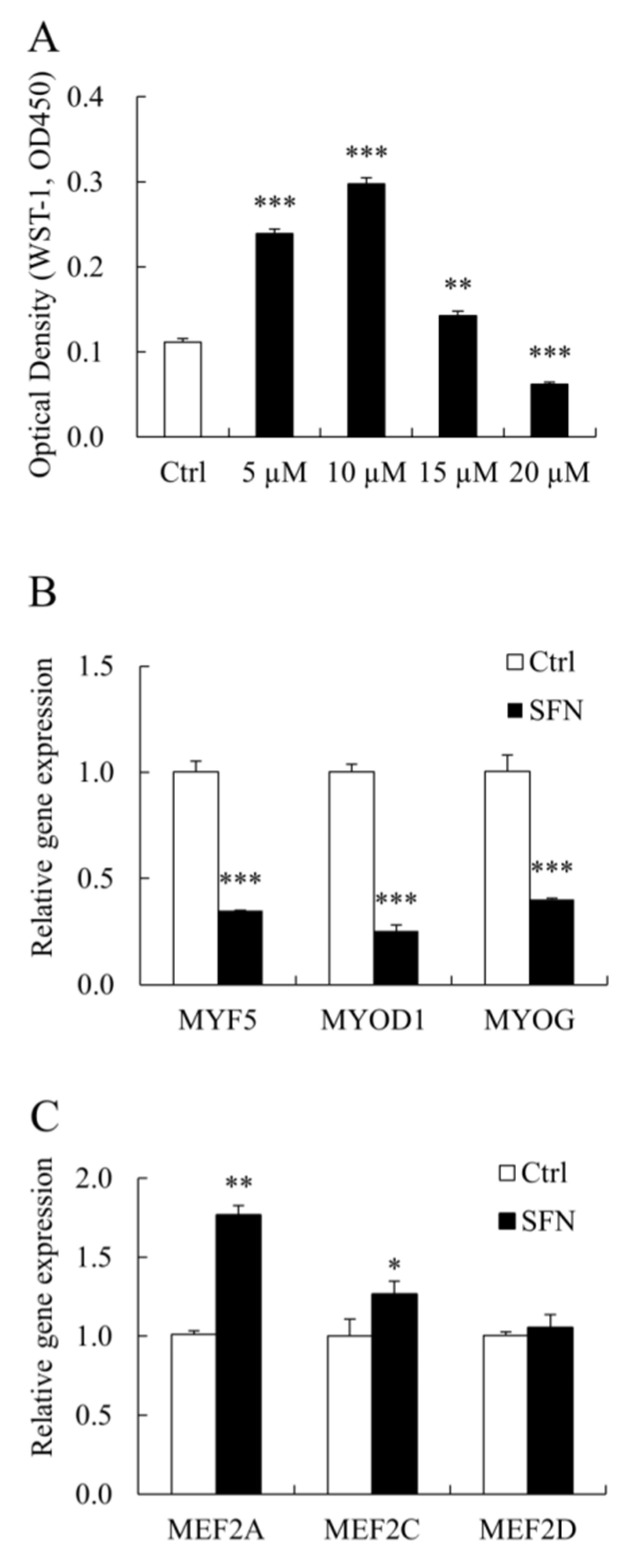
SFN modulated PSC proliferation and myogenic transcription factors (TFs). (**A**): The viability of PSCs treated with Ctrl or SFN at different concentrations (5, 10, 15, or 20 μM). (**B**,**C**): qRT-PCR results of PSC proliferation and differentiation associated genes in Ctrl and SFN (10 μM) groups. Data were shown as the mean ± SE and assays were done in triplicates. * *p* < 0.05, ** *p* < 0.01, *** *p* < 0.001.

SFN influenced PSC proliferation in a dose-dependent manner. SFN at 5, 10, and 15 μM increased PSC proliferation, whereas SFN at 20 μM had the reverse effect (Figure 2A). As a consequence, 10 μM SFN was used in the following study. SFN inhibited the expression of *MYOD1* mRNA, which is consistent with our prior findings [4]. SFN also reduced the levels of *myogenic factor 5* (*MYF5*) and *Myogenin* (*MYOG*) mRNA expression (Figure 2B). In PSCs treated with SFN, however, MEF2 family members’ (*MEF2A* and *MEF2C* but not *MEF2D*) mRNA levels were elevated (Figure 2C).

### 3.2. SFN Inhibited HDAC Activity and Elevated Global Histone Acetylation Level

**Figure 3:**
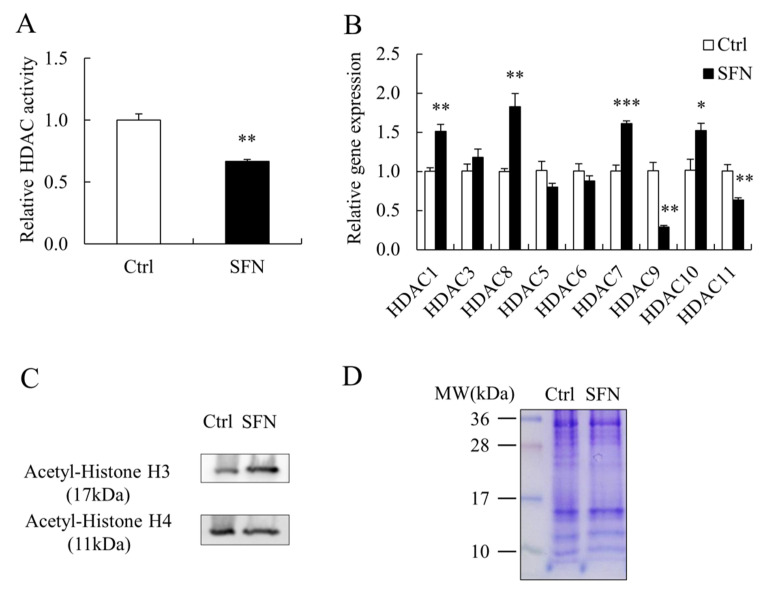
SFN increased global histone acetylation in PSCs. (**A**): The HDAC activity in Ctrl and SFN treated PSCs; (**B**): HDAC family members’ relative mRNA expression levels; (**C**,**D**): Western blot results of acetylated histone H3 and H4 in PSCs treated with Ctrl and SFN, and Coomassie-Brilliant Blue staining as loading control. Data are shown as the mean ± SE and assays were done in triplicates. * *p* < 0.05, ** *p* < 0.01, *** *p* < 0.001.

SFN dramatically reduced the activity of HDAC in PSCs (Figure 3A) and had an effect on HDAC family members’ mRNA levels. There are four classes (I–IV) of HDAC family members in the classical HDAC family. *HDAC1* and *HDAC8* of Class I, as well as *HDAC7* and *HDAC10* of Class II, had higher mRNA levels in the SFN group than those in the Ctrl group, while *HDAC9* of Class II and *HDAC11* of Class IV had lower mRNA levels (Figure 3B). Meanwhile, global acetylated histone H3 and H4 levels were significantly higher in SFN-treated PSCs (Figure 3C), with Coomassie-Brilliant Blue staining serving as a loading control (Figure 3D).

### 3.3. SFN Increased SMAD7 Expression in PSCs

**Figure 4:**
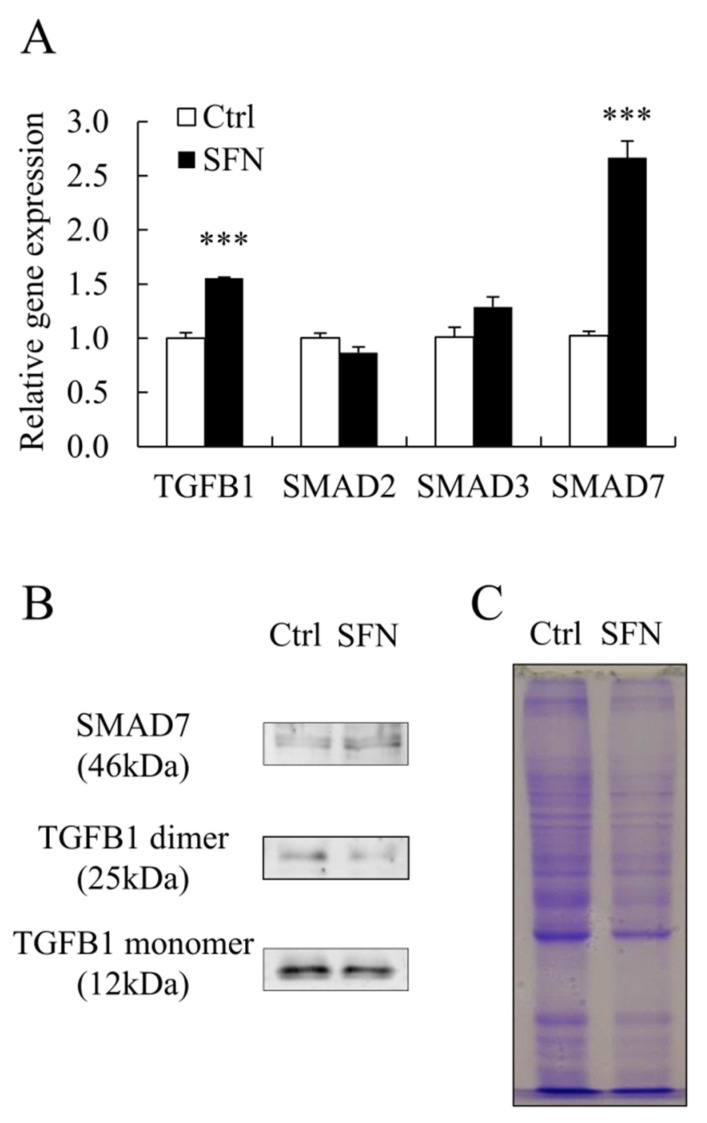
The expression of *SMAD7* was upregulated by SFN treatment. (**A**): Relative mRNA expression levels of *TGFB1*, *SMAD2*, *SMAD3*, and *SMAD7*; (**B**,**C**): The protein levels of *TGFB1* and *SMAD7* with Coomassie-Brilliant Blue staining as loading control. Data are shown as the mean ± SE and assays were done in triplicates. *** *p* < 0.001.

SFN treatment increased the abundance of *SMAD7* mRNA and protein, as well as the level of *TGFB1* mRNA (Figure 4A,B), while decreasing the amount of *TGFB1* protein (Figure 4B). SFN treatment had no effect on *SMAD2* or *SMAD3* mRNA levels (Figure 4A).

### 3.4. SFN Altered the Expression of TFs and miRNAs Involved in Regulating SMAD7

**Figure 5:**
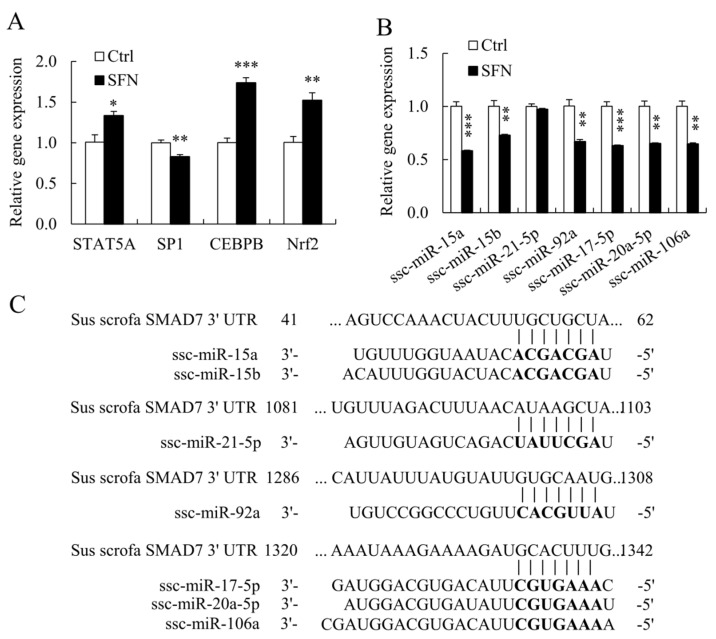
TFs and miRNAs regulating *SMAD7* were remodeled by SFN treatment. (**A**): Relative mRNA expression levels of porcine *SMAD7* TFs; (**B**): Relative expression levels of porcine miRNAs targeting *SMAD7*; (**C**): The alignments of porcine miRNAs and corresponding binding sites in *SMAD7* 3′ UTR. Data are shown as the mean ± SE and assays were done in triplicates. * *p* < 0.05, ** *p* < 0.01, *** *p* < 0.001.

SFN enhanced *STAT5A*, *CEBPB*, and *Nrf2* mRNA levels in PSCs but reduced *SP1* mRNA expression (Figure 5A), whereas PROMO [27] and TFSEARCH [28] demonstrated that all these TFs bind to the porcine *SMAD7* promoter.

Multiple miRNAs target *SMAD7* in humans, including *hsa-miR-15b*, *hsa-miR-20a*, *hsa-miR-21*, *hsa-miR-92a*, and *hsa-miR-106b* [11,29]. TargetScan also predicts that *ssc-miR-15a* and *ssc-miR-17-5p* will target *SMAD7* [20]. Figure 5C showed the alignments of porcine miRNAs and their associated binding sites in the porcine *SMAD7* 3′ UTR. SFN inhibited the expression of *ssc-miR-15a*, *ssc-miR-15b*, *ssc-miR-92a*, *ssc-miR-17-5p*, *ssc-miR-20a-5p*, and *ssc-miR-106a* in porcine miRNAs (Figure 5B).

### 3.5. SFN Elevated Histone H4 Acetylation Level at SMAD7 Promoter

**Figure 6:**
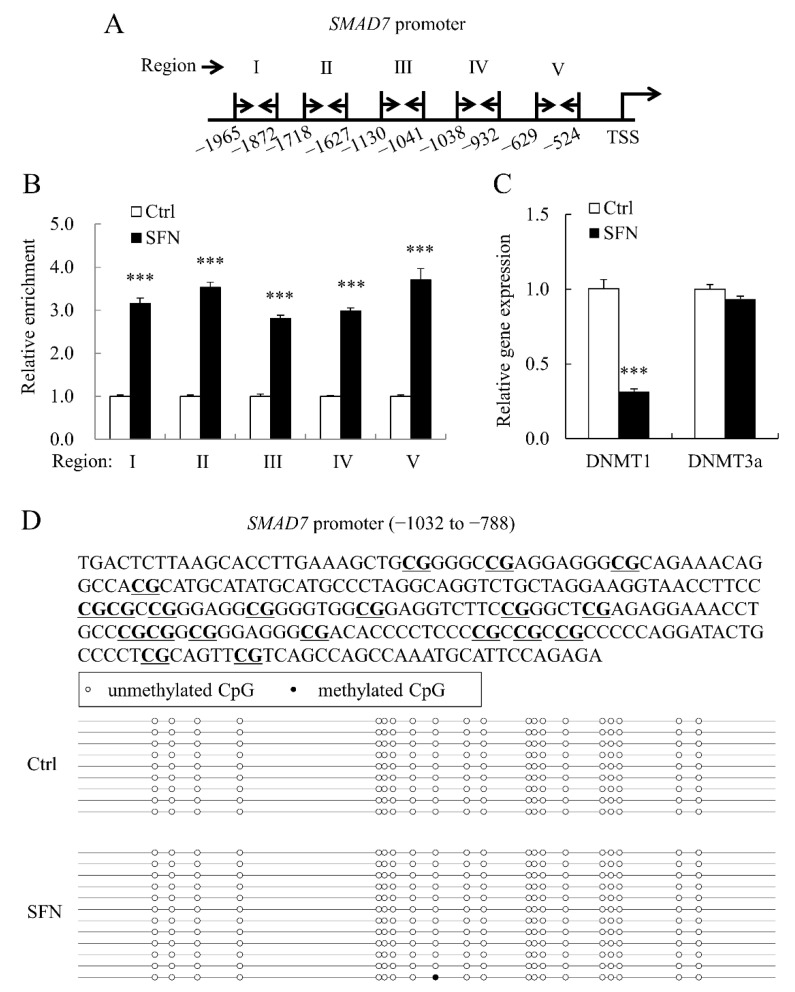
Histone H4 acetylation in porcine *SMAD7* promoter was enriched by SFN. (**A**): The schematic map shows the locations of primer sets (forward primer: right arrow and reverse primer: left arrow) used in ChIP-qPCR; (**B**): Relative enrichment fold change of histone H4 acetylation of five regions across porcine *SMAD7* promoter; (**C**): DNMT1 and DNMT3a relative mRNA expression levels in Ctrl and SFN groups; (**D**): The methylation status of CpG island (−1032 to −788 bp) in the *SMAD7* promoter. CpG sites are highlighted with underline in the upper panel. Data are shown as the mean ± SE and assays were done in triplicates. *** *p* < 0.001.

As previously stated, SFN raised global histone acetylation levels in PSCs. We also used ChIP-qPCR to look at the degree of histone H4 acetylation in the porcine *SMAD7* promoter. To amplify five areas throughout the 2 kb promoter of porcine *SMAD7*, five pairs of primers (Table 1) were utilized for ChIP-qPCR (Figure 6A). The levels of histone H4 acetylation at promoter areas were dramatically increased by SFN (Figure 6B).

The mRNA level of DNA methyltransferase 1 (*DNMT1*) was decreased by SFN (Figure 6C), which is consistent with our previous findings [4]. Bisulfite sequencing was used to investigate the methylation status of the porcine *SMAD7* promoter. All CpG sites in the *SMAD7* promoter region studied (Figure 6D) were unmethylated and SFN administration had no effect on this pattern.

## 4. Discussion

Satellite cells have been a hot topic of research since their discovery in 1960 [4]. Satellite cells are essential for muscle development, maintenance, and regeneration since they are the skeletal muscle’s stem cells. SFN dramatically boosted PSC proliferation and *SMAD7* mRNA and protein expression.

SFN is abundant in cruciferous vegetables such as broccoli and Brussels sprouts. We discovered that SFN can control the growth of PSCs. PSC proliferation was boosted by SFN at 5, 10, and 15 µM, whereas PSC proliferation was hindered by SFN at 20 µM. In mice, satellite cell population in the muscle fiber of extensor digitorum longus is enlarged with SFN as diet supplement [14]. SFN has similar dosage-dependent effects on human mesenchymal stem cells (MSCs) [30], where low-dose SFN (0.25 and 1 µM) promotes MSC proliferation as an antioxidant but high-dose SFN (20 µM) kills MSCs. The transformation of SFN from a mitogen to a cytotoxin implies that the underlying processes induced by low and high levels of SFN are likely to vary. SFN mitogenic impact on PSCs was the focus of the current study.

MYOD transcription is stimulated in active satellite cells, and MYF5 protein begins to accumulate. Until the differentiation program is started, MYOG is not transcribed [31]. SFN inhibited the myogenic pathway of PSCs by inhibiting *MYF5*, *MYOD1*, and *MYOG* mRNA levels. Similar findings were reported in research using the mouse myoblast C2C12 cell line, in which SFN (2 µM) reduced the protein abundances of MYOD and MYOG [32]. Although SFN raised MEF2 mRNA levels in PSCs, low levels of MYOD1 and MYOG reduced MEF2’s transcriptional capacity. As a result, SFN inhibits myogenic differentiation.

The MEF2-dependent transcription is inhibited by class IIa HDACs, which interfere with the myogenic program [33]. Increased Class IIa HDAC expression suppresses MEF2C expression and reduces myoblast differentiation [34]. The reduced activity of HDACs in PSCs caused by SFN might alleviate the suppression of MEF2 and MEF2-dependent myogenic gene transcription by class IIa HDACs. In the current study, SFN has a variety of impacts on HDAC members. SFN, for example, reduces HDAC3 and HDAC6 protein levels in HeLa cells [35], but has no effect on nuclear HDAC abundance in human embryonic kidney 293 cells. It was also discovered that HDAC members’ mRNA levels did not respond evenly to SFN exposure in our study.

SFN dramatically enhanced global histone H3 and H4 acetylation levels in PSCs. This is consistent with the majority of earlier studies [12]. There are, however, a few works that make different observations. SFN treatment has little effect on global histone acetylation in RT4 and UMUC3 human bladder cancer cells, despite the fact that HDAC activity is dramatically reduced [36].

SFN was observed to lower *TGFB1* protein levels while increasing *SMAD7* mRNA and protein levels in this study, which was associated with increased PSC proliferation. Excess TGF-β slows satellite cell multiplication and decreases satellite cell stemness [37]. HDAC inhibitors increase SMAD7 expression in fibroblasts [38] and breast cancer cells [39]. *SMAD7* deficiency in mice causes reduced satellite cell proliferation and differentiation, as well as a reduction in muscle mass [7].

We examined the expression of multiple important TFs for pig *SMAD7*. SFN raised the levels of *STAT5A*, *CEBPB*, and *Nrf2* mRNA, while decreasing the levels of SP1 mRNA. SFN promotes Nrf2 signaling, which leads to the induction of *SMAD7* [40]. In addition to transcriptional regulation, various miRNAs inhibit *SMAD7* expression at a post-transcriptional level [11,29], and SFN downregulates multiple miRNAs targeting porcine *SMAD7*.

Bisulfite sequencing was also employed to determine the methylation status of the porcine *SMAD7* promoter. According to our findings, all CpG sites in the *SMAD7* promoter examined area were unmethylated, and this state was unaffected by SFN therapy. SFN, on the other hand, greatly enhanced the local histone H4 acetylation level of the *SMAD7* promoter in PSCs. Enriched histone acetylation is normally linked with active promoters.

## 5. Conclusions

**Figure 7:**
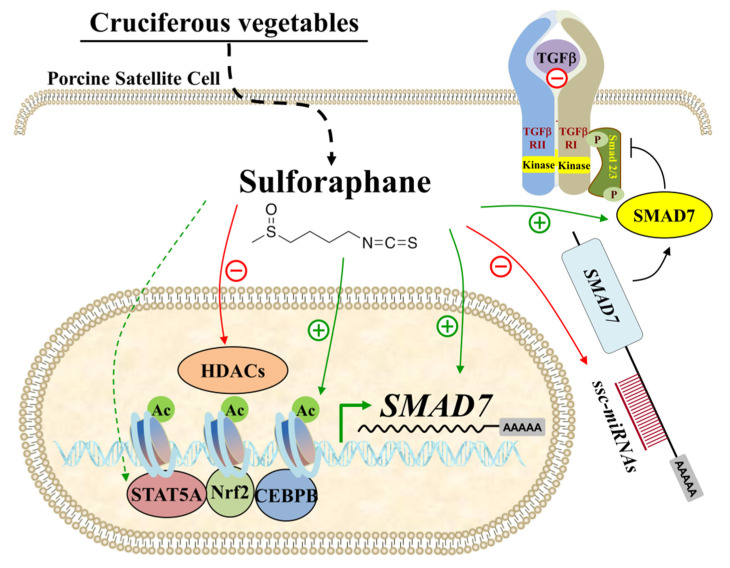
SFN upregulated *SMAD7* expression in PSCs. SFN enriched the histone H4 acetylation level in the *SMAD7* promoter and suppressed the expression of miRNAs targeting SMAD7, which led to increased mRNA and protein abundance of *SMAD7* in PSCs.

In conclusion, SFN promotes the proliferation of PSCs and inhibits myogenic differentiation. Our findings reveal that SFN influences the expression of myogenic regulator factors and MEF2. Furthermore, SFN upregulates *SMAD7* expression in PSCs via histone acetylation and the miRNA route (Figure 7). These findings update our understanding of how skeletal muscle stem cell activity interacts with dietary supplements. Our work indicates that SFN supplementation in the diet can help with muscle growth and repair through satellite cell proliferation enhancement.

## Figures and Tables

**Table 1:** The sequences of primers used in this study.

Purpose	Gene	Sequence (5′—3′)	Size (bp)	GenBank Accession Number
qRT-PCR for mRNA expression	MYF5	F: AGACGCCTCAAGAAGGTCAA		NM_001278775
		R: TCCTGCAGGCTCTCAATGTA	128	
	MYOD1	F: TGCAAACGCAAGACCACTAA		NM_001002824
		R: GCTGATTCGGGTTGCTAGAC	127	
	MYOG	F: CAGTGAATGCAGTTCCCACA		NM_001012406
		R: GGTGAGGGAGTGCAGATTGT	130	
	MEF2A	F: TGATGCGGAATCATAAAATCG		NM_001099698
		R: GCACCAGTAGTTCCAACCAAA	358	
	MEF2C	F: CGAGATACCCACAACACACG		NM_001044540
		R: CGCTTGACTGAGGGACTTTC	175	
	MEF2D	F: TCACTGCAGTTCAGCAATCC		XM_021089672
		R: AGGCCAGGAGACACACTGTT	128	
	HDAC1	F: GGAAATCTATCGCCCTCACA		XM_013999116
		R: AAACACCGGACAGTCCTCAC	157	
	HDAC3	F: CAACCAGGTGGTGGACTTCT		NM_001243827
		R: GCAGAGGGATGTTGAAGCTC	152	
	HDAC5	F: AGATGCACTCCTCCAGTGCT		XM_021066892
		R: GGATGATGGCAAATCCATTC	102	
	HDAC6	F: ATGGACGGGTATTGCATGTT		XM_003360315
		R: GCGGTGGATGGAGAAATAGA	168	
	HDAC7	F: CGTCCCCTACAGAACTCTCG		XM_021092604
		R: TCAGGTTGGGCTCAGAGACT	146	
	HDAC8	F: GGTGACGTGTCTGATGTTGG		XM_021080459
		R: AGCTCCCAGCTGTAAGACCA	165	
	HDAC9	F: AACTGAAGCAACCAGGCAGT		XM_021102482
		R: CCCAACTTGTCCCAGTGAGT	149	
	HDAC10	F: TCCATCCGAGTACCTTCCAC		XM_021091335
		R: GGCTGCTATGGCCACACTAT	179	
	HDAC11	F: GACAAGCGCGTGTACATCAT		XM_021069384
		R: AGGTTCCTCTCCACCTTCGT	143	
	TGFB1	F: CGTGCTAATGGTGGAAAGCG		XM_021093503
		R: AGAGCAATACAGGTTCCGGC	122	
	SMAD2	F: GCAATCTTTGTGCAGAGCCC		NM_001256148
		R: ACACGGCTTCAAAACCCTGA	157	
	SMAD3	F: GCTGGACGACTACAGCCATT		NM_214137
		R: TGTGGTTCATCTGGTGGTCG	140	
	SMAD7	F: CCAACTGCAGACTGTCCAGA		XM_005659454
		R: CAGGCTCCAGAAGAAGTTGG	106	
	STAT5A	F: GAGGTGCTGAAGAAGCATCA		NM_214290
		R: GGCTTCAGATTCCACAGGTT	200	
	SP1	F: TGCAGCAGAATTGAGTCACC		XM_005652567
		R: ACTGCTGCCACTTTGTTCCT	180	
	CEBPB	F: GCTTGAACAAGTTCCG		NM_001199889
		R: CAAGAAGACCGTGGATAAGC	209	
	Nrf2	F: GTGCCTATAAGTCCCGGTCA		XM_013984303
		R: ATGCAGAGCTTTTGCCCTTA	108	
	DNMT1	F: GCGGGACCTACCAAACAT		NM_001032355
		R: TTCCACGCAGGAGCAGAC	133	
	DNMT3a	F: CTGAGAAGCCCAAGGTCAAG		NM_001097437
		R: CAGCAGATGGTGCAGTAGGA	238	
	HPRT1	F: AACCTTGCTTTCCTTGGTCA		NM_001032376
		R: TCAAGGGCATAGCCTACCAC	150	
qRT-PCR for miRNA expression	ssc-miR-15a	F: TAGCAGCACATAATGGTTTGT	-	MIMAT0007753
	ssc-miR-15b	F: TAGCAGCACATCATGGTTTACA	-	MIMAT0002125
	ssc-miR-17-5p	F: CAAAGTGCTTACAGTGCAGGTAG	-	MIMAT0007755
	ssc-miR-20a-5p	F: TAAAGTGCTTATAGTGCAGGTA	-	MIMAT0002129
	ssc-miR-21-5p	F: TAGCTTATCAGACTGATGTTGA	-	MIMAT0002165
	ssc-miR-92a	F: TATTGCACTTGTCCCGGCCTGT	-	MIMAT0013908
	ssc-miR-106a	F: AAAAGTGCTTACAGTGCAGGTAGC	-	MIMAT0002118
Bisulfite sequencing PCR	SMAD7-Bis	F: TGATTTTTAAGTATTTTGAAAGTTG		
		R: TCTCTAAAATACATTTAACTAACTAAC	245	
ChIP-qPCR	Region I	F: TATGCCTCATGCACAGCACC		
		R: CCCATGCACAGGGAAAGACA	93	
	Region II	F: ATTGCAGCCTCTGTGGCTTA		
		R: GACCTAGGGATGCCAAGCAG	91	
	Region III	F: TGGTCCTTTGCCCTACCAAC		
		R: ATCCCCTTAGCCTGCGTTTT	89	
	Region IV	F: ACTCTCTGACTCTTAAGCACCT		
		R: AGGTTACCTTCCTAGCAGACCT	106	
	Region V	F: CGGTCCAGTCCGGTGTAAAT		
		R: CGTTTTGCCTTAAAGGCCCTG	105	

## Data Availability

The data presented in this study are available on request from the corresponding author.
